# Direct currents stimulate carbonate mineralization for soil improvement under various chemical conditions

**DOI:** 10.1038/s41598-020-73926-z

**Published:** 2020-10-12

**Authors:** Dimitrios Terzis, Patrick Hicher, Lyesse Laloui

**Affiliations:** Soil Mechanics Laboratory, Swiss Federal Institute of Technology Lausanne (EPFL), EPFL ENAC IIC LMS Station 18, 1015 Lausanne, Switzerland

**Keywords:** Civil engineering, Geochemistry, Bioinspired materials

## Abstract

The present study integrates direct electric currents into traditional calcium carbonate mineralization to investigate electrochemical interactions and the subsequent crystalline growth of CaCO_3_ bonds in sand. A specific line of focus refers to the effect of three chemical reactive species involved in the stimulated geo-chemo-electric system, namely CaCl_2_, Ca(CH_3_COO)_2_ and Ca(CH_3_CH_2_(OH)COO)_2_. By altering treatment conditions and the applied electric field, we capture distinctive trends related to the: (i) overall reaction efficiencies and distribution of CaCO_3_ crystals is sand samples; (ii) promotion of CaCO_3_ mineralization due to DC (iii) crystallographic and textural properties of mineralized bonds. The study introduces the concept of EA-MICP which stands for Electrically Assisted Microbially Induced Carbonate Precipitation as a means of improving the efficiency of soil bio-cementation compared to traditional MICP-based works. Results reveal both the detrimental and highly beneficial effects that electric currents can hold in the complex, reactive and transport processes involved. An interesting observation refers to the “doped” morphology of CaCO_3_ crystals, which precipitate under electric fields, validated by crystallographic analyses and microstructural observations.

## Introduction

Electro-chemical applications have been brought into focus as a potential way of consolidating soils mainly targeting soft clays with first attempts reported as early as 1967 when Fetzer^1^ described a system of steel bars placed in soft clay up to a depth of 10 m to act as electrodes and treat an area of 200 m^2^. After 103 days of treatment, a total settlement of the targeted zone equal to 50 cm was achieved at a power consumption equal to 17 kWh per m^3^ of clay stabilized. The result was increase in the soil’s undrained strength from 8.8 to 108 kPa and a water content reduction from 31% to jut 7%. Since then progress has been reported in both in the laboratory and field-scale^2–4^. Systems to integrate electrokinetics (EKs) into other geotechnical systems have been also reported especially around the use of electric vertical drains (EVDs)^5,6^. Decontamination or dewatering applications^7,8^ appear as the most suitable candidates for the application of EKs given the absence of any other alternative technique to remove pollutants from contaminated sites. The involved technical complexity and economic costs are believed to hinder a more mainstream adoption of EKs into traditional geotechnical systems which are currently designed based on more traditional solutions, such as consolidation via preloading.

During the past decade, bio-chemical treatment for improving soils is emerging^9–12^ as an alternative solution to more traditional ground stabilization applications such as those based on cement, fly ash or petroleum-based resins. Microbially Induced Carbonate Precipitation (MICP) represents the most predominant and well-studied version of bio-inspired technologies in geotechnical and geo-environmental engineering. The present study aims to shed light on the critical role applied EKs can hold in MICP and present a novel integration of an EKs system into ground bio-stabilization practice. The reactive solutions involved in MICP are rich in microbes which carry enzymatic activities responsible for decomposing urea into carbon dioxide and ammonia which solvate into bicarbonate (CO_3_^2−^) and ammonium (NH_4_^+^) ionic species. CO_3_^2−^ reacts with calcium Ca^2+^ which is available in the groundwater or introduced into the system. The treatment solutions are therefore electrolytes of high ionic strength. We postulate that the application of direct currents and the consequent electrically induced flow of solutes would impact significantly the fate and evolution of the consolidation process through the formation of stable, CaCO_3_ minerals inside the porous networks of geo-materials. More precisely, applied EKs enhance electrical motion forces that drive the distribution of either dissolved ions, charged inorganic and organic particles. Electromigration stands-out as the most efficient electrically induced motion force of dissolved ions. The electrical force **F** = *qE*, with q being the charge of the considered species and **E** the electric field across the specimen, distributes the ions according to the distribution of electric potential.

The overall complex effects of applied electric fields on the formation, functioning and degradation of microbial communities are understood with respect to the targeted bioremediation application, such as that referring to oil contaminated sites^13^. As far as MICP is concerned, few relevant attempts to induce MICP under applied electric fields have been reported. Keykha et al.^14–17^ present experiments where applied electric currents on *Pasteurii* micro-organisms yield electrophoretic migration which depends on their zeta potential, the pH of the environment (which is higher at cathode end and lower at the anode end) as well as on the possibility to enhance the rate of transport through sandy clay specimens when the imposed electric current increases^14^. The complexity of the involved phenomena lies into the succession of injections which are rich in dissolved ionic species of opposite electric charge. For example, Ca^2+^ can be introduced close to the anode, while CO_3_^2−^ ions, which originate from microbially hydrolysed urea, are introduced close to the cathode. This strategy has been shown to result into repellent forces that drive cations towards the cathode and vice versa. Overall, a good rate of calcification is postulated when reactive species cross paths. This was demonstrated for soft clay (kaolinite) treatment through EKs transport (electromigration and electroosmosis)^15^. Specific focus is given on the role of applied EKs on handling unwanted by-products, result of MICP. These are residual nitrogen species (dissolved ammonium and ammonia gas) and chlorides. Residual ammonium can potentially become a source of environmental contamination^16^ and therefore its removal through electromigration is a promising way of addressing the fate of this element which otherwise appears as a major hurdle to overcome towards an efficient upscaling of MICP.

Overall, electrically assisted crystallization in continuous flows, for transforming amorphous species into structured, crystalline patterns is known in the literature for protein^18^ and mineral synthesis^19^. Some of the reported benefits are the ability to control the spatial location of nucleation or to increase the product number, size and quality. Specifically, for calcium carbonate species, the role of applied electric currents or that of altering chemical surface conditions on the growth patterns has been the focus of several studies. The incorporation of soluble additives, such as polyacrylic acid^20^ or Polylisine^21^ has been proven to induce the formation of polarized calcite substrates with distinctive, elongated structures attributed to the electric conditions.

Based on the current state of knowledge we postulate that different Ca^2+^ sources will alter the chemical surface conditions. By altering between field-free and applied electric field conditions for the same Ca^2+^ used, we investigate whether distinctive crystalline morphologies yield. The goal is to determine the detrimental and beneficial effects of direct currents on carbonate mineralization for the complex process of MICP targeting ground improvement applications. These effects are captured and presented through laboratory testing on sand columns for varying applied electric fields and chemical conditions with the aim to suggest an efficient EA-MICP approach which enhances the desired mineralization process and therefore the properties of the engineered geo-material.

## Materials and methods

The study introduces three alternative, Ca^2+^-rich reactive sources, for investigating the efficiency in inducing MICP under applied DCs. Based on the current state of knowledge, most of the MICP-focused studies use CaCl_2_ as a reactive species, mainly because of its lower cost, availability and high solubility^11^. CaCl_2_ is an inorganic salt with well-known electrolytic reactions which affect significantly the processes involved in MICP. To illustrate these effects, we consider that when using CaCl_2_ as a calcium source, Cl^−^ anions undergo oxidative reaction (Eq. 1) at the anode

1 $$2{\text{Cl}}^{ - } \to {\text{Cl}}_{2} \left( {\text{l}} \right) + 2{\text{e}}^{ - }$$

due to local low pH conditions. The production of active chlorine results into the formation of hypochlorous acid is described in Eq. 2:

2 $${\text{Cl}}_{2} \left( {\text{l}} \right) + {\text{H}}_{2} {\text{O}} \to {\text{HOCl}} + {\text{H}}^{ + } + {\text{Cl}}^{ - }$$

which is in equilibrium with hypochlorite at pH around 7.5. Among oxidants, hypochlorite holds a strong disinfecting effect and serves as the principal disinfectant in bleach water. Hypochlorite causes electrophilic attacks on amino and thiol groups of proteins^22^. Microorganisms which are equipped with plasma membranes are also vulnerable to hypochlorous acid which has the ability to penetrate into the lipid bilayer of living cells and destroy them. We investigate the detrimental effect of applied DCs when using CaCl_2_ as a MICP reactant and we further extend MICP treatment using calcium acetate (CaAc_2_) and calcium lactate (CaL_2_) as reactive species. This choice is done based on the solubility of these species as well as due to the fact that acetates and lactates represent organic, soft bases which act as pH buffer to balance the production of acids. Despite the lower solubility of these latter (Table 1), the absence of Cl^−^ and of its detrimental role, allows for determining the effects of applied DC on the crystallization process.

**Table 1:** Calcium sources used in the study of EA-MICP.

Ca^2+^ source	Solubility (g/100 ml at 20 °C)	Dissolved Ca^2+^ (mol/l)
CaCl_2_	74.5	6.71
Ca(C_2_H_3_O_2_)_2_	34.7	2.19
CaL_2_	4.08	0.19

MICP is applied using *Sporosarcina Pasteurii* cells on sand columns batch experiments following the treatment process previously reported in Terzis and Laloui^23^. Monitoring of the reaction is done using Electrical conductivity (EC) analysis coupled with Ion Chromatography (IC) measurements to detect ionic species and evaluate the depletion of reactants (Ca^2+^) and production of ammonia (NH_4_^+^) to compare EA-MICP with conventional MICP.

**Figure 1:**
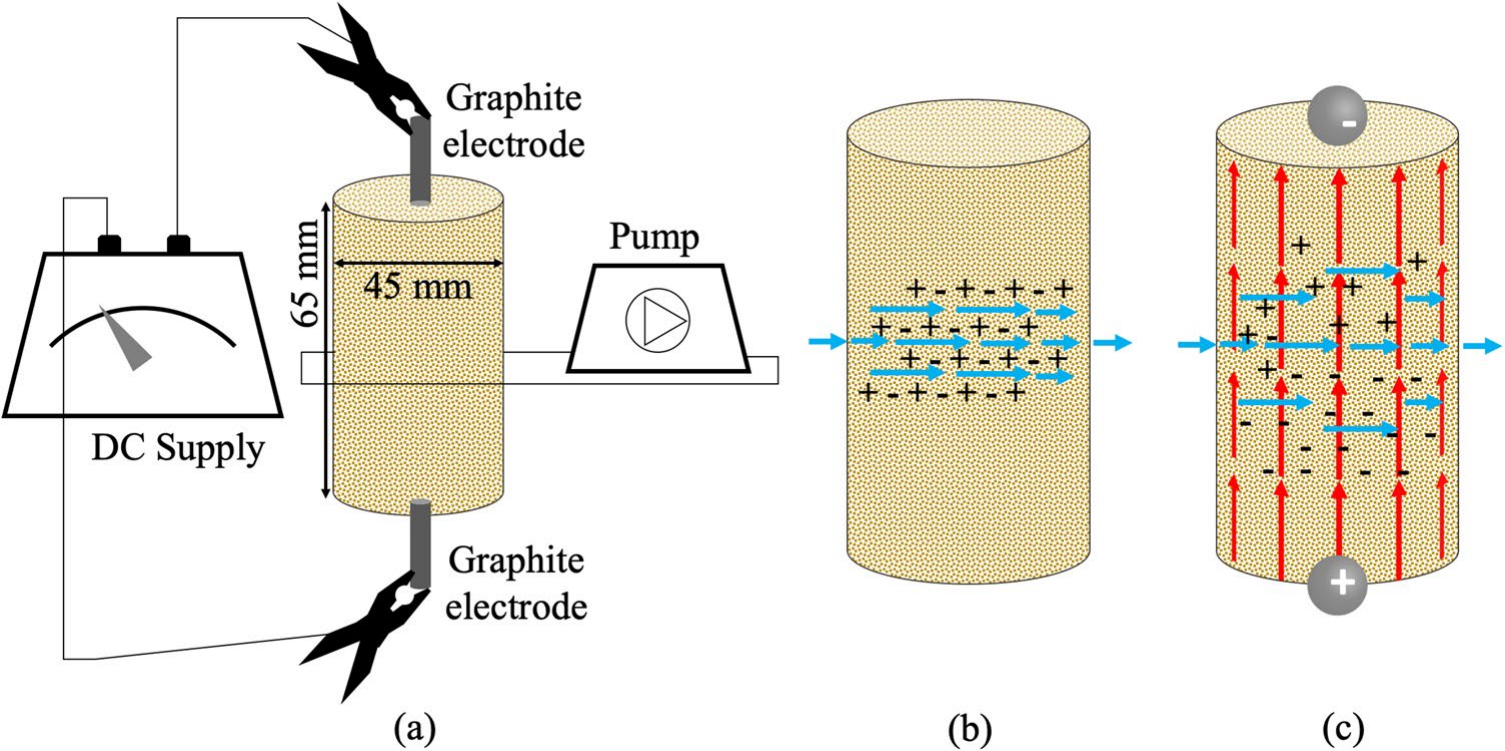
(**a**) schematic representation of the setup used to induce EA-MICP in sand columns; (**b**) blue arrows indicate the direction of the flow of reactive solutes in traditional MICP using the setup of (**a**) without application of DC; (**c**) red arrows indicate the applied electric field via a cathode (−) and anode (+) supplied with DC for the same infiltration system shown in (**a**) and (**b**).

Direct currents in column experiments are applied using the setup of Fig. 1. Electrodes are placed at the top and bottom of the column while the inflow and outflow ports (Fig. 1b), for introduction of the electrolytes, are placed in the middle of the column. Hydraulic flow is therefore transversal to the electric potential field induced by the applied DC (Fig. 1c). The polarity of the field can be fixed or alternate between batches during EA-MICP. Batches contain calcium and urea and are provided on a daily basis to the column. The mass of sand in each column is 170 g for a total volume of 100 cm^3^ which yields a void ratio of approximately 0.4. the base material is a natural sand collected from Lake Léman, Switzerland, with is properties shown in Table 2.

**Table 2:** Base material properties.

Parameter	
Mean particle size (mm)	0.6
Minimum void ratio (emin)	0.48
Maximum void ratio (emax)	0.66
Coefficient of curvature (cc)	1.04
Uniformity coefficient (cu)	4.4
CaCO_3_ content (%)	16

A fixation solution of high salinity is firstly supplied to the columns to pre-treat the soil and enhance bacterial attachment. More precisely, fixation solutions are composed of CaL_2_, CaAc_2_ or CaCl_2_ at a concentration of 0.25 M. A pre-treated sample with deionized water acts as the control sample. To quantify bacteria attachment, 50 mL of the fixation solution, i.e. 2.5 times the pore volume, was flushed through the columns at an injection rate of 10 mL/min. The cell attachment is quantified by measuring the optical density (OD) at 600 nm of the outflow solution and comparing it with that of the injected bacterial batch, which has a value of OD equal to 0.905. More precisely, 1 mL of the solution that flows out of the columns is collected after 0.5, 1 and 1.5 pore volumes have been circulated. During both treatments, an electric tension of 10 V is applied. The intensity of the generated DC varies between 1, 15, 30 and 80 mA for the control H_2_O, CaL_2_, CaAc_2_ and CaCl_2_, respectively. These fixation solutions display electroconductivities of 0.02, 5, 8 and 24 mS/cm respectively.

The effect of direct currents on the electrolysis of urea was investigated individually. A urea-rich solution was electrolyzed and the for 24 h and the resulted suspension was mixed with CaCl2. Urea hydrolysis is not found to occur in the tested electrocatalysis configuration. This is believed to be due to the use of graphite electrodes which explains this poor engagement of urea into the system. With this respect, metal catalysts such as nickel are found to determine the electrolysis of urea according to Boggs et al.^24^. For inducing mineralization of CaCO_3_ the reactive solutions are supplied in various concentrations, with equimolar concentrations of Ca^2+^ and urea. The concentration of the CaL_2_ was 0.25 M and that of CaAc_2_ was 0.5 M. In order to investigate the effect of an applied DC on calcite distribution, 5 and 15 V electrical tensions are applied. The distance between the electrodes is 5 cm, i.e. they penetrate the column for 7.5 mm on both ends of the sand column, which results into the generation of electric fields of 1 and 3 V/cm. One could consider herein that the current flow intensity reported in literature, reaches 27,700 V/cm or 28 V for a 18 cm distance in a laboratory setup described by Lee^5^.

Results are presented with respect to the EC variation, NH_4_^+^ and Ca^2+^ concentration in outflows, as well as with respect to calcite distribution across the bio-cemented columns. Finally, Scanning Electron Microscopy (SEM) observations on the texture of bio-cemented sand under EA-MICP illustrate the morphology and fabric of mineralized calcite and compare it to that under conventional MICP. Additionally, crystallographic patterns are identified through XRD analyses. Results are provided for the base material (pure sand) and compared to those obtained post-MICP and post-EA-MICP to determine the CaCO_3_ phase which precipitates under the applied DCs and interpret results with respect to the Ca^2+^ source used. To understand results in the best possible light, we combine calcite measurements, XRD crystallography, SEM observations and micro-Computed Tomography. This latter is carried out to evaluate qualitatively the porosity of samples post-MICP and post-EA-MICP.

## Results

### Cell attachment

**Figure 2:**
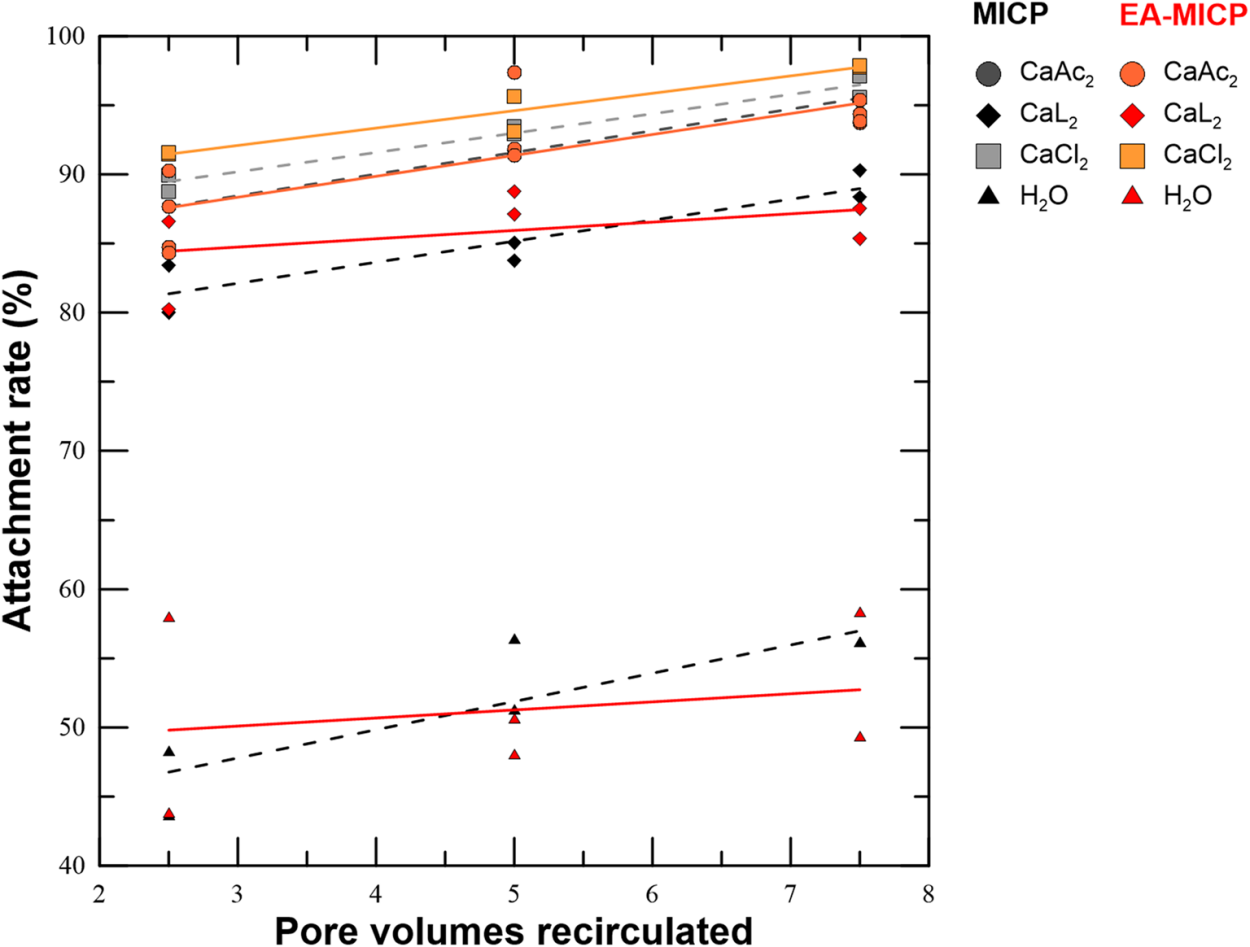
Cell attachment rate with respect to recirculated volume through the sand column for MICP and EA-MICP, and three different fixation solutions (CaCl_2_, CaAc_2_, CaL_2_) compared to no fixation solution (H_2_O).

Figure 2 reveals that when the sand column is pre-treated with a fixation solution, before injecting the bacterial solution, the resulted cell attachment reaches 90%. In comparison, attachment rate varies between 43 and 57% when cells are flushed through the sand column which was previously water saturated. Continuous injection until 1.5 pore volumes reveals slight increase in the attachment rate with respect to the injection time. CaL_2_ yields the lowest attachment rate when compared to CaCl_2_ and CaAc_2_. The applied electric field does not seem to influence the attachment rate however it is still unknown how the cell distribution across the column is affected. Post-treatment CaCO_3_ measurements are expected to answer this uncertainty. Overall it is demonstrated that pre-conditioning the soil with a saline solution enhances the attachment efficiency regardless of the injected volume or applied electric field due to electrostatic bounding of the Ca^2+^ between the negatively charged surfaces of the sand grains and the bacteria cells.

### Sand column experiments

**Figure 3:**
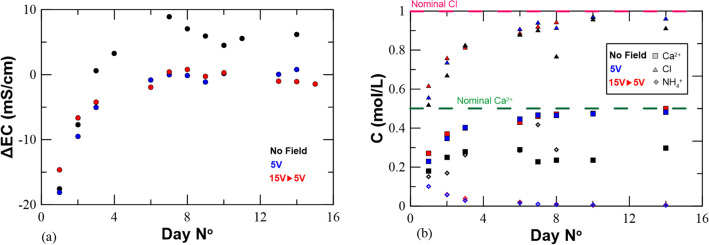
Monitoring CaCl_2_-basedMICP and EA-MICP by (**a**) electric conductivity measurements and (**b**) ion chromatography analyses.

Electroconductivity (EC) measurements provide an indirect way of monitoring ionic species production/consumption (NH_4_^+^ and Ca^2+^). Differential EC (ΔEC) between the injected, parent solution and the collected outflow (Fig. 3a) shows that under applied electrical current, EC varies significantly compared to the field-free condition after 3 days. ΔEC settles to approximately + 5 mS/cm in the field-free case, whereas in the case of applying electric potentials of 5 V and varying potential between 15 and 5 V, no further difference in EC is recorded. ΔEC measurements are compared to the more robust technique of ionic chromatography (IC) analyses which quantify ionic concentration. Under applied DC no NH_4_^+^ is detected after 6 days and Ca^2+^ concentrations remain at the level of the parent solution’s concentration (0.5 M). Contrary, for the field-free case, NH_4_^+^ production continues over time and Ca^2+^ consumption is observed (Fig. 3b). This is attributed to the detrimental effect of the produced HOCl which hinders the enzymatic activity and leads to CaCO_3_ dissolution at the anodic end due to the production of an acidic front. Regarding Cl^−^ ion concentration, the same trends are observed in both cases with concentrations reaching the nominal parent solution concentration after 6 days.

**Figure 4:**
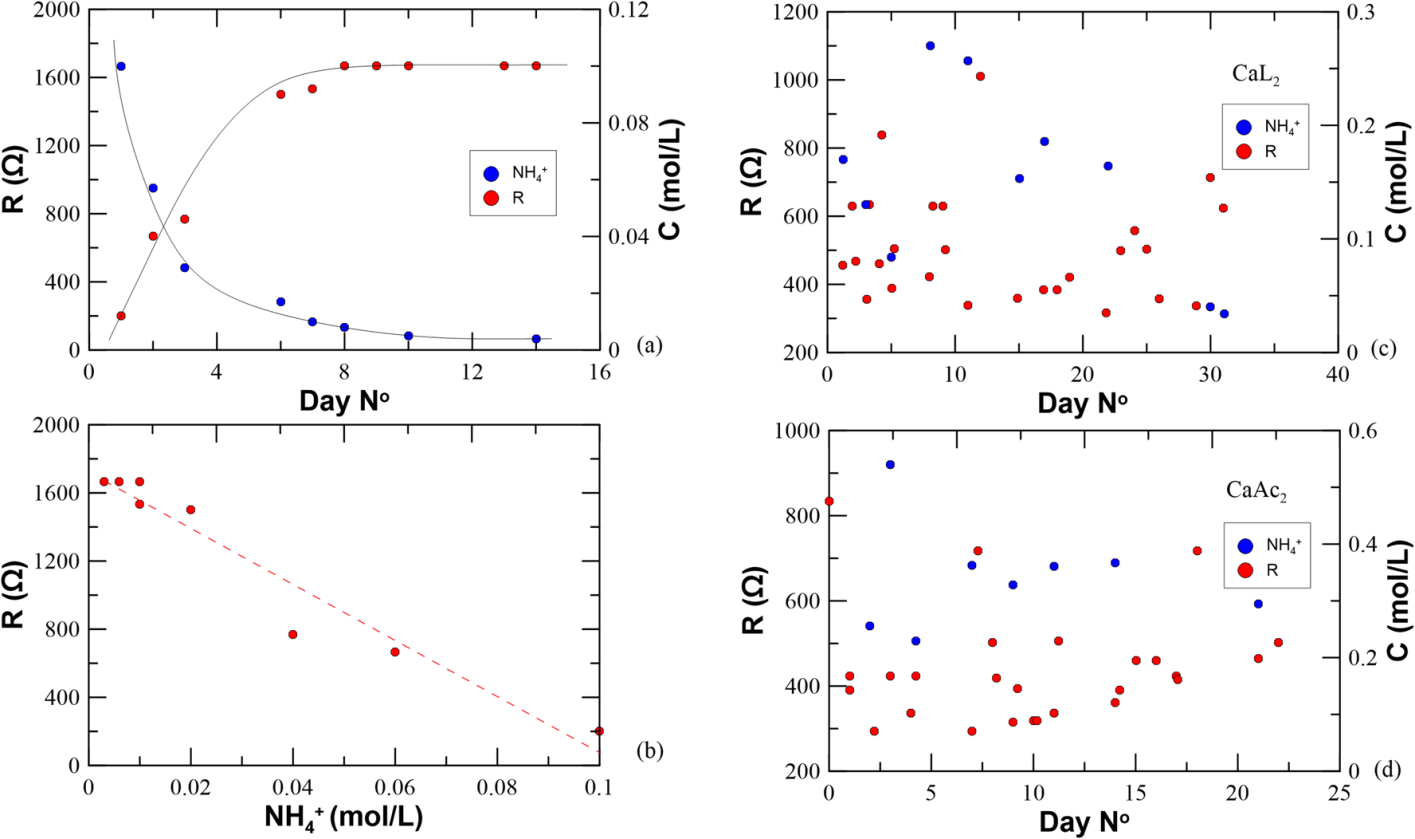
Plots of the recorded electrical resistivity over time periods of applied EA-MICP under 5 V in: (**a**) CaCl_2_ medium with (**b**) the relationship between the sample’s resistance and NH_4_^+^content, (**c**) CaL_2_ medium and (**d**) CaAc_2_ media.

Figure 4 illustrates the NH_4_^+^ concentration in the outflow solution when CaCl_2_ is used as a reactive species (a and b) under 5 V of applied DC. Results reveal a linear correlation between the increased solution’s resistivity (the reciprocal of EC) with decreasing NH_4_^+^ content in the outflow solution. This reflects the electrosynthesis occurring in the chloride medium which is responsible for the degradation of the bacteria cells which results into inhibition of the enzymatic activity and therefore to no further production of NH_4_^+^. Looking at the recorded resistivity of the samples treated with CaL_2_ and CaAc_2_ under 5 V of applied DC (Fig. 4c,d respectively) no relationship with the ammonium concentration can be extracted. This reflects ongoing electrochemical reactions, which however do not hinder bacterial activity and where NH_4_^+^ production yields a resistivity decrease and the competing mechanism of CaCO_3_ mineralization results into resistivity increase. This explains why an overall quasi-null ΔR is observed.

**Figure 5:**
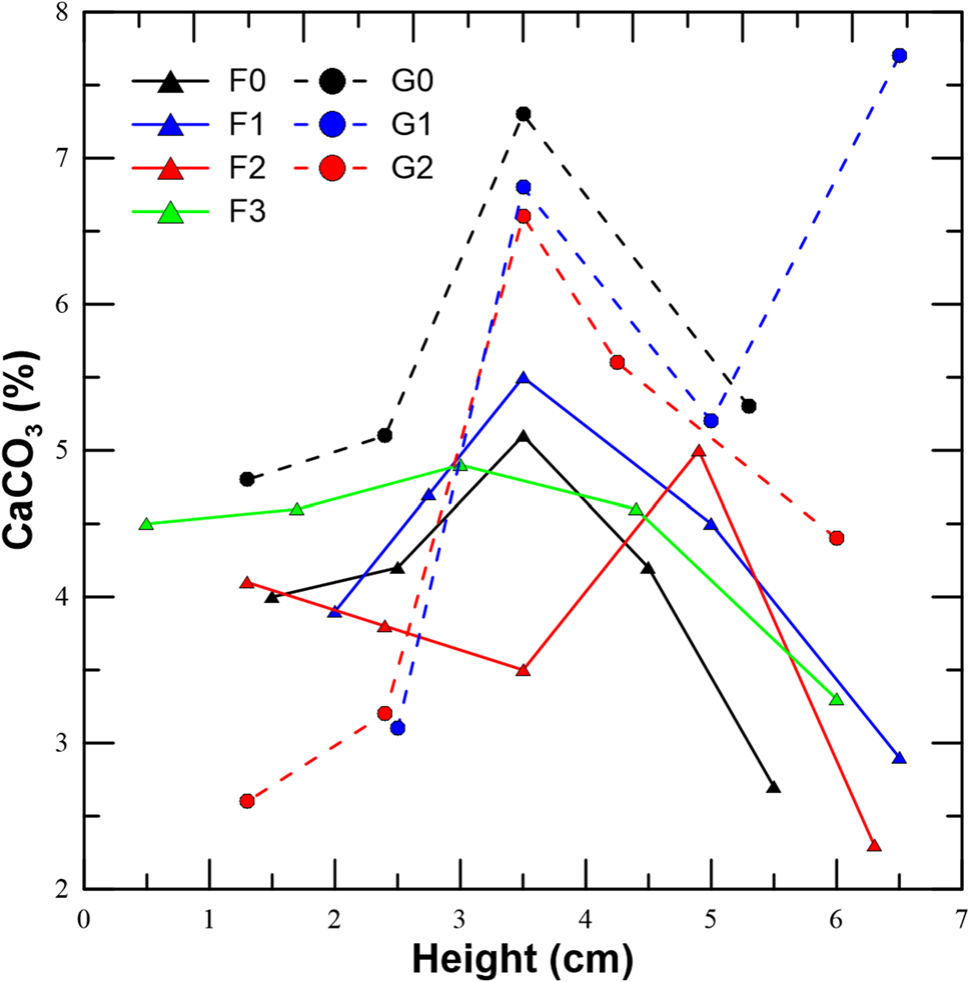
Calcite content distributions across the bio-cemented columns’ height under no-field (0) and three applied fields of 5, 10, 15 V for samples bio-cemented using CaL_2_ (F series) and CaAc_2_ (G-series).

The mineralized CaCO_3_ distribution across the columns is further illustrated in Fig. 5 for the samples produced using CaL_2_ and CaAc_2_ under two applied voltages and compared to the case of no applied field. The F series (CaL_2_ samples) in Fig. 5 shows that both F_0_ (no field) and F_1_ (5 V field of fixed polarity) samples display a similar trend with a maximum content observed in the middle section, where the reactive solution enters and flows out of the specimen. However, we observe that under an applied 5 V electric field (F_1_), the distribution of CaCO_3_ shifts slightly towards higher contents both in the middle and towards the cathode end of the sample, where calcium is attracted. The same behaviour is observed for F_2_ and F_3_ samples which yield an extension of the cemented blocks towards the cathode with higher calcite contents than for the field-free (F_0_) condition. The most homogenous distribution is yielded for F_3_ sample, bio-cemented under alternating polarity of 15 V. This much improved homogeneity across the sample’s height is due to the successive electromigration of Ca^2+^ upwards or downwards, depending on the polarity of the applied electric field (see Fig. 1).

The efficiencies of produced NH_4_^+^ and that of depleted Ca^2+^ are shown in Table 3. The efficiency is both expressed as the ratio of the measured NH_4_^+^ in the outflow over the theoretical NH_4_^+^ production, should urea had completely reacted and as the ratio of the Ca^2+^ present in the outflow over the initial Ca^2+^ of the reactive inflow solution. It should be noted that CaAc_2_-treated samples were provided with 0.5 M concentration compared to 0.25 M for CaL_2_. Therefore, lower efficiency does not necessarily reflect lower calcite contents. G samples reveal a different CaCO_3_ distribution pattern with the exception of G_0_ (field-free) case where the same variations are observed with respect to F_0_. G_1_ and G_2_ samples both display a local peak in calcite content in the middle section and a very strong depletion towards both the the anodic and cathodic ends. As far as the G_1_ sample is concerned, the drop observed in calcite content in the middle is compensated by the increase towards the cathode, due to electromigration of Ca^2+^ ions which result in strong depletion in the anode region where supersaturation of the solution drops, resulting in reduced calcium carbonate precipitation. At 15 V applied voltage of fixed polarity, lower calcite content is yielded for the cathodic end while the rest of the distribution remains identical to that of 5 V fixed voltage. The sample G3 under alternating polarity did not yield sufficient bio-cementation and therefore its distribution is not shown.

**Table 3:** Variation of: ureolysis efficiency rate (based on NH_4_^+^ IC); CaCO_3_ efficiency (based on Ca^2+^ IC) and pH for samples F (CaL_2_-treated) and G (CaAc_2_-treated) during 17 batches of MICP (samples 0) and EA-MICP (samples 1, 2, 3) treatment.

Ca^2+^ source	Sample	Polarity	Infiltrations 1–9		Infiltrations 10–17		Total		pH
		(V, +/−)	NH_4_^+^	Ca^2+^	NH_4_^+^	Ca^2+^	NH_4_^+^	Ca^2+^	
CaL_2_	F0	0	0.29	0.18	0.37	0.16	0.33	0.17	7.9
	F1	5, bottom/top	0.36	0.19	0.32	0.16	0.34	0.18	8.2
	F2	15, bottom/top	0.30	0.17	0.25	0.14	0.28	0.15	8.1
	F3	15, alternating	0.28	0.16	0.34	0.17	0.31	0.17	7.8
CaAc_2_	G0	0	0.14	0.11	0.37	0.14	0.24	0.12	7.7
	G1	5, bottom/top	0.11	0.08	0.26	0.09	0.18	0.08	7.9
	G2	15, bottom/top	0.16	0.09	0.25	0.09	0.20	0.09	7.8
	G3	15, alternating	0.11	0.09	0.21	0.06	0.16	0.08	7.8

**Figure 6:**
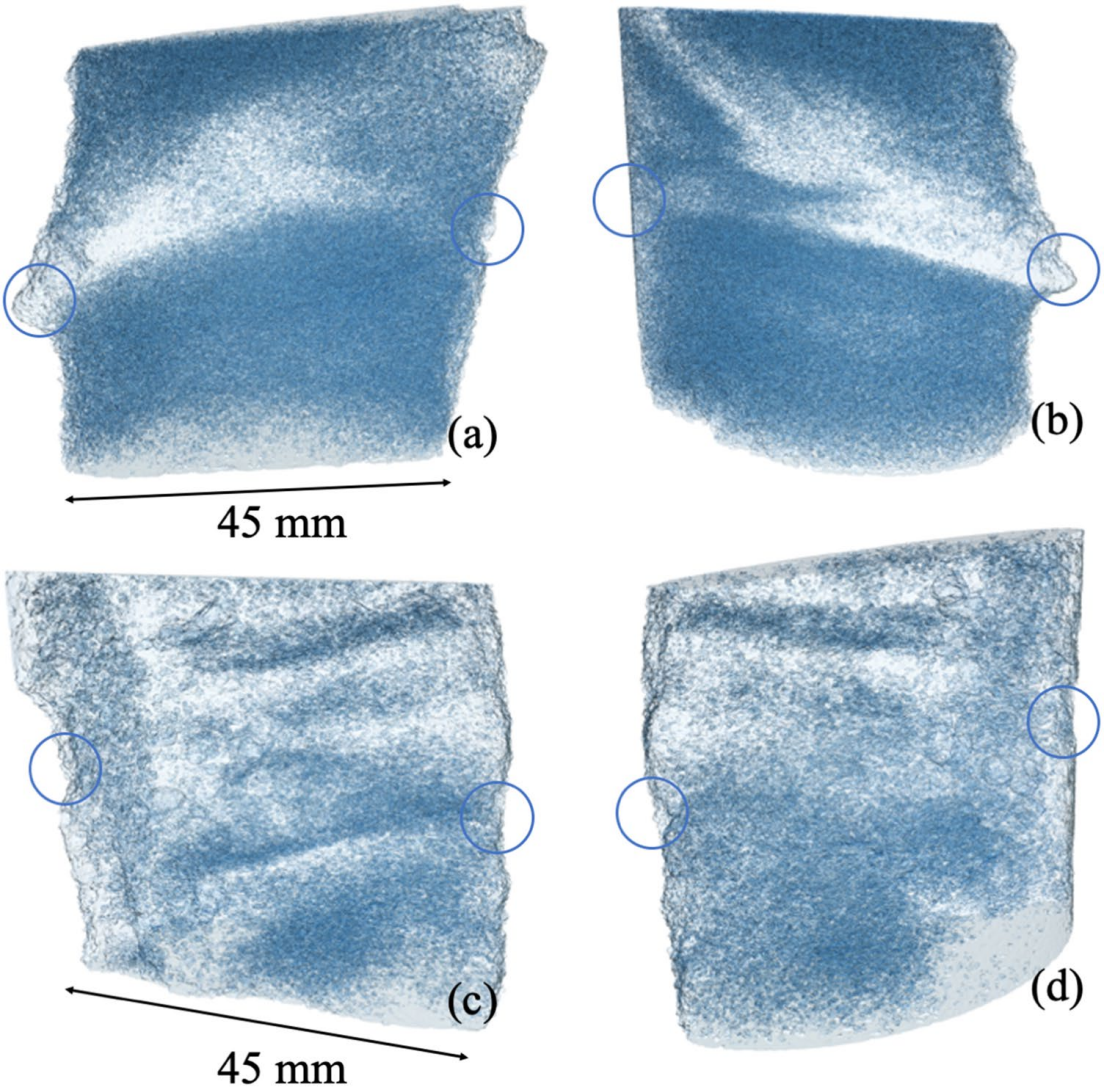
Micro-CT scans of samples G0 (MICP) and G1 (EA-MICP) of Fig. 5; the interconnected porosity is shown in blue and zones of increased mass of CaCO_3_ are identified in in white.

Figure 6 illustrates the micro-CT analyses carried out on samples G0 of Table 3 (a and b), treated under MICP using the configuration of Fig. 1, and sample G1 produced under EA-MICP (c and d). Results present the mapping of the interconnected porosity of the samples (blue) and are obtained after image processing using Avizo^25,26^ at a voxel resolution of 24.8 μm. Analyses reveal zones which are heavily bio-cemented (white). A clear zone of strong bio-cementation is seen in a and b which connects the injection ports (circles) of the sand column treated under MICP. Under the applied 5 V DC field, the relatively heavier zones of CaCO_3_ mineralization are more well-spread across the sample, and especially on the upper end where the cathode is connected, and calcium is attracted.

### Crystallography of MICP and EA-MICP

**Figure 7:**
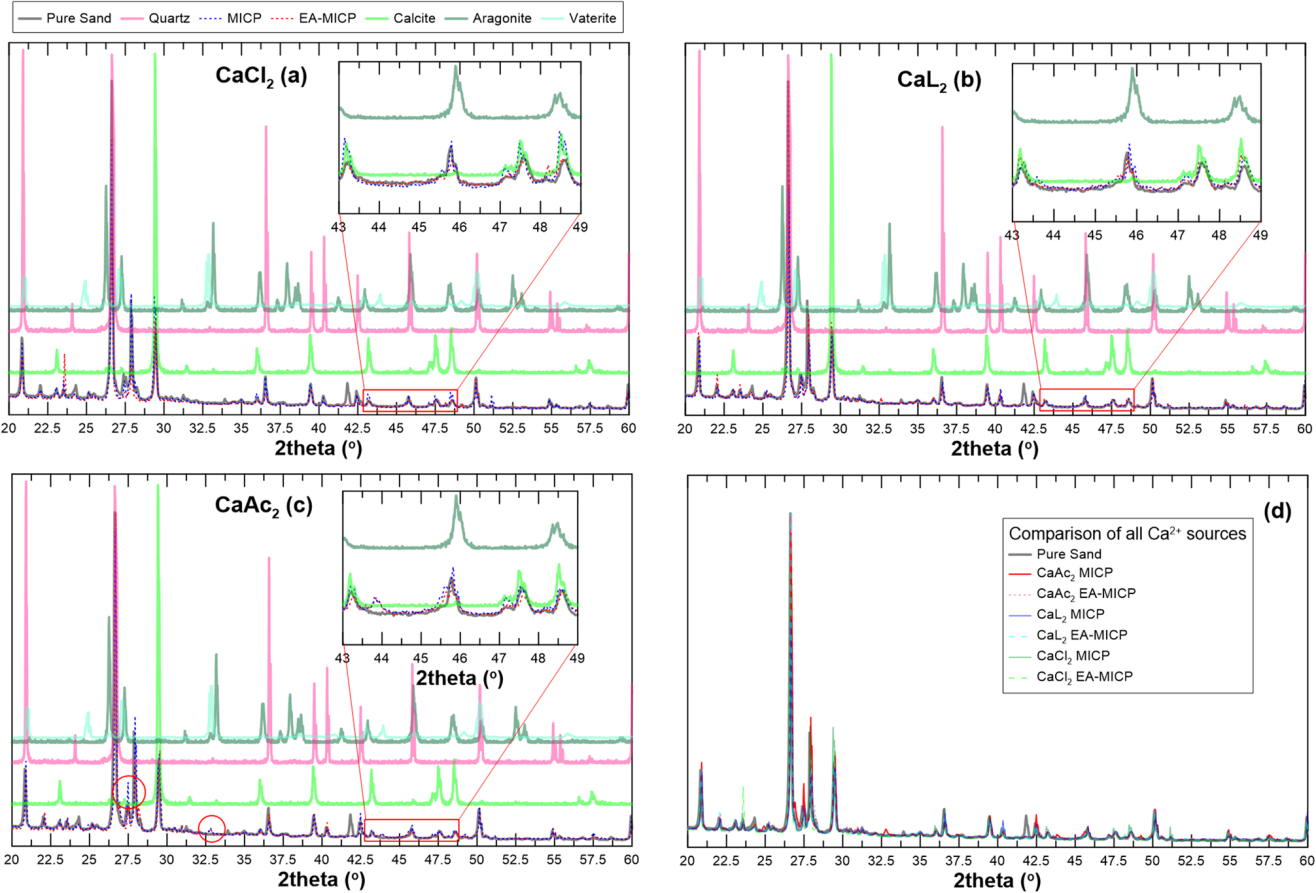
XRD patterns of MICP (no applied DC) and EA-MICP (applied 5 V of DC) samples obtained after treating pure sand with CaCl_2_ (**a**), CaL_2_ (**b**) and CaAc_2_ (**c**); (**d**) comparison of all samples obtained under treatment with three calcium sources; Inlets in (**a**, **b**, **c**) narrow down the focus in the range between 43–49 degrees where calcite peaks 202, 204 and 108 are expected, the predominant 104 peak at ~ 29.5 degrees is shown in Fig. 8; circles in c represent notable peaks due to CaAc_2_-based MICP and EA-MICP which correspond to aragonite and vaterite.

**Figure 8:**
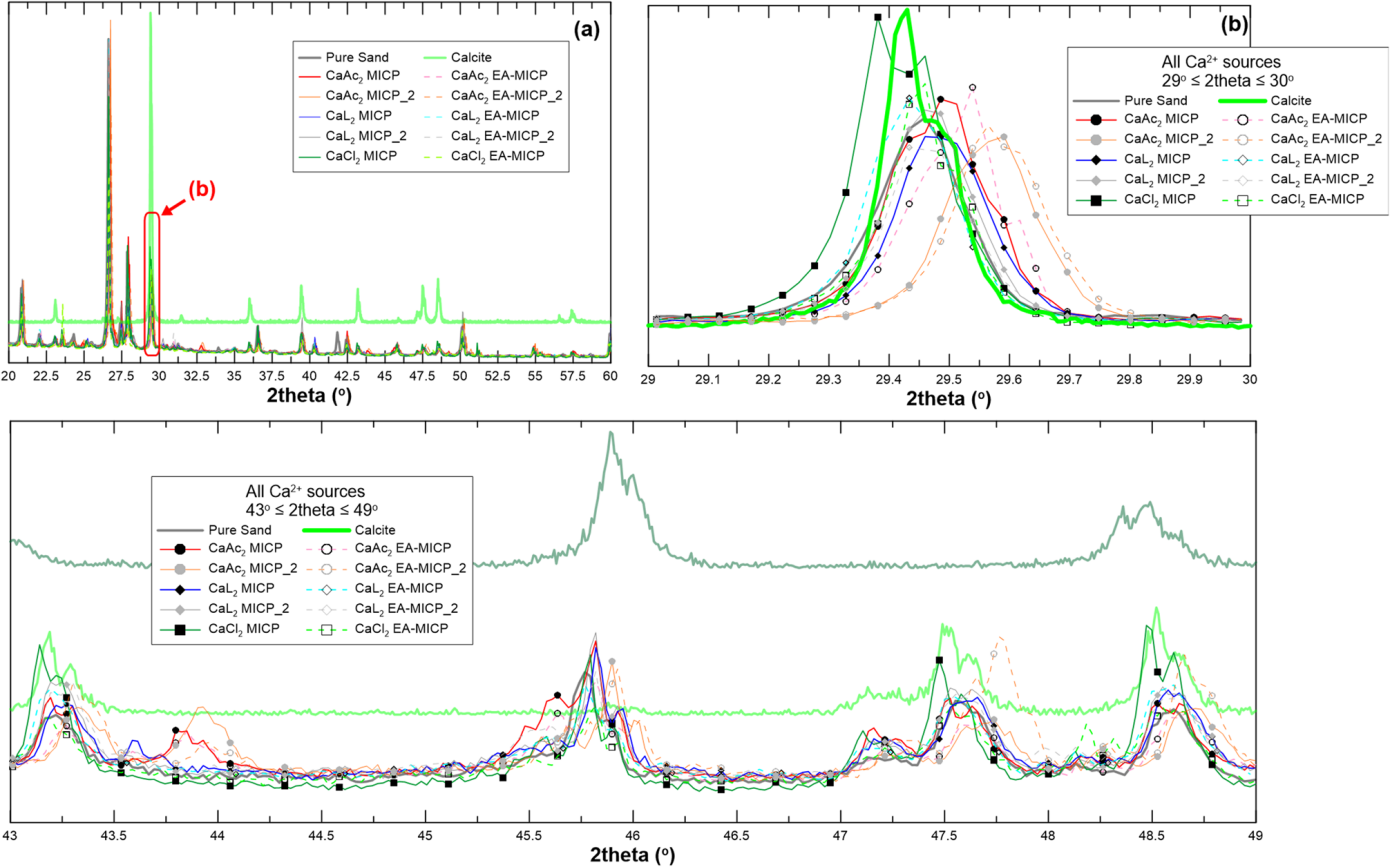
XRD patterns of ten samples of MICP (no applied DC) and EA-MICP (applied 5 V of DC) samples obtained after treating pure sand with CaCl_2_, CaL_2_, and CaAc_2_; (**a**) comparison of all ten samples; (**b**) narrowing down the range of interest around the predominant 104 calcite peak; (bottom) narrowing down the range of interest around the 43–49 2theta degree range where calcite peaks 202, 204 and 108 are expected.

The diffractograms of the pure sand, and of the same material subjected to MICP and EA-MICP using CaCl_2_, CaL_2_ and CaAC_2_ are shown in Fig. 7. All diffractograms are compared with patterns which correspond to pure SiO_2_, pure calcite, pure aragonite and vaterite diffractograms. Patterns for the CaCO_3_ phases are obtained from Zhou et al.^27^. The focus is put on comparing the spectra obtained for the MICP and EA-MICP treatment for all chemical conditions applied (i.e. alternative calcium sources). A specific line of focus is put on the 2thetha region between 29 and 30 degrees (Fig. 8) where the calcite 104 phase, i.e. the most predominant calcite peak, is expected. To facilitate the presentation of results, a second zone of specific interest in resented in in Fig. 8 between 2theta values of 43 and 49. Figure 8 summarizes patterns from 10 bio-cemented samples in pairs of MICP and EA-MICP treatments, with one pair for CaCl_2_ and two pairs for CaL_2_ and CaAc_2_ respectively.

The pure natural sand has an intrinsic calcite content of 16% (Table 2) which reflects on its XRD diffractogram (predominant calcite 104 peak at approximately 29.5 degrees of 2theta). When, CaCl_2_ is used as reactive species, notable shifts in the diffractogram are observed around 2theta values of 43, 47.5 and 49 which correspond respectively to the calcite peaks 202, 204 and 108 with the latter overlapping with the aragonite 041 peak^27^ (Fig. 7a, inlet). The increased intensity of these peaks reflects larger presence of calcite especially for the MICP case. EA-MICP with CaCl_2_ as reactant does not yield the same peak intensity with the MICP treatment which reflects the inhibition of the mineralization process which was captured from the results of chemical analyses presented in “Cell attachment” and “Sand column experiments” sections. Similar conclusions are drawn through narrowing the focus in the region of 2theta values between 29 and 30. Herein, CaCl_2_-based MICP yields a wider 2theta range than natural sand. We postulate that a shift of the corresponding 2theta angle reflects crystalline imperfections while a wider range of 2theta is believed to be the result of overgrown crystallites or of a wider crystalline size distribution. To further evaluate this hypothesis, SEM observations will be presented in the following section.

XRD patterns for the CaL_2_ treatment samples are analysed and yield double diffraction peaks for the range of 2theta between 43 and 49 degrees. These latter diffraction patterns are more pronounced both for MICP and EA-MICP in terms of intensity. In Fig. 8, the EA-MICP sample based on CaL_2_ yields increased intensity for the 104 and 202 calcite peaks compared to the MICP treatment and to pure sand. Contrarily, the sole improvement captured for sample CaL_2_ EA-MICP_2 is the wider peak for calcite 202. SEM observations are expected to further shed insight into the textural and surface characteristics of precipitated nuclei.

CaAc_2_ yields significant shifts in the captured XRD patterns. More precisely, Fig. 7c reveals notable new peaks around 27.5 and 33 degrees of 2theta. These peaks are more pronounced for the MICP treatment when compared to the EA-MICP and reflect higher contents of aragonite and vaterite compared to pure sand. Figure 8 reveals that the predominant 104 calcite peak around 29.5 degrees is more pronounced both in terms of intensity and also in terms of shifting towards higher 2theta values for the EA-MICP treatment. This wider range is believed to reveal increased crystalline size distribution. This shift is more pronounced for sample CaAc_2_ EA-MICP_2 which yields higher CaCO_3_ contents with respect to the other two samples.

### Microstructure of MICP and EA-MICP

**Figure 9:**
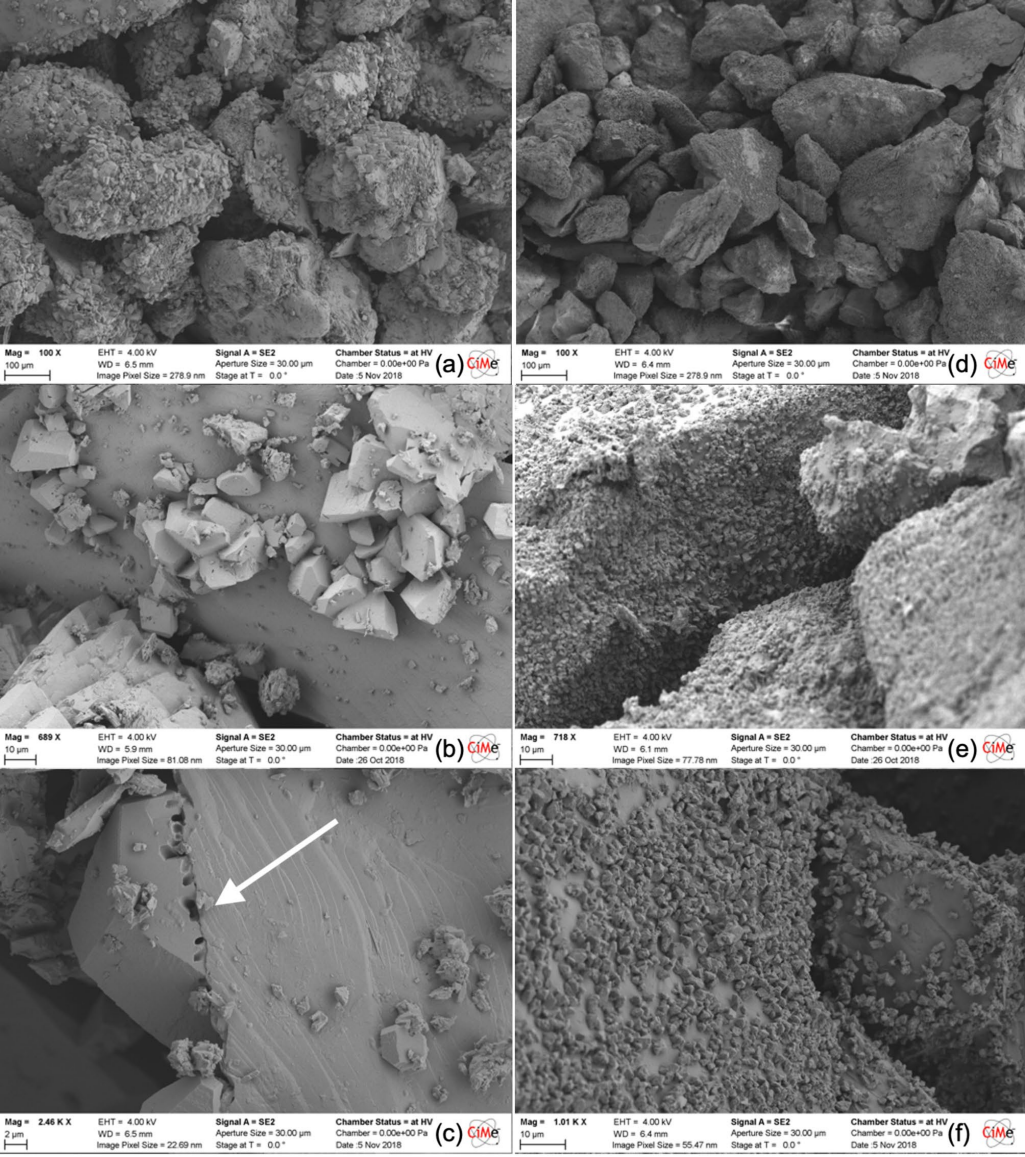
SEM observations on the surface of bio-cemented sand samples which were obtained after MICP (**a**–**c**) and EA-MICP (**d**–**f**) using CaCl_2_ as reactive species.

Results on the fabric characteristics of MICP and EA-MICP bio-cemented sand are presented with respect to the three alternative Ca^2+^ sources used. CaCl_2_ treatment yields calcite bonds which can be seen in Fig. 9 under 3 different magnifications. MICP and EA-MICP samples are characterized by the presence of rhombohedral calcite particles (104 phase). Crystals exhibit larger sizes for the MICP treatment, reaching 10 μm, which validates the lower intensity in the crystallographic peaks captured in Figs. 7a and 8a. Further, traces of bacteria cells are seen in MICP-treated samples (arrow in Fig. 9c). No traces are seen in any of the images 9d to 9f. which suggest complete degradation of the bacteria cells due to hypochlorination. These results further validate the trends observed in “Sand column experiments” and “Crystallography of MICP and EA-MICP” sections regarding the drawback of applying DC to CaCl_2_-based MICP.

**Figure 10:**
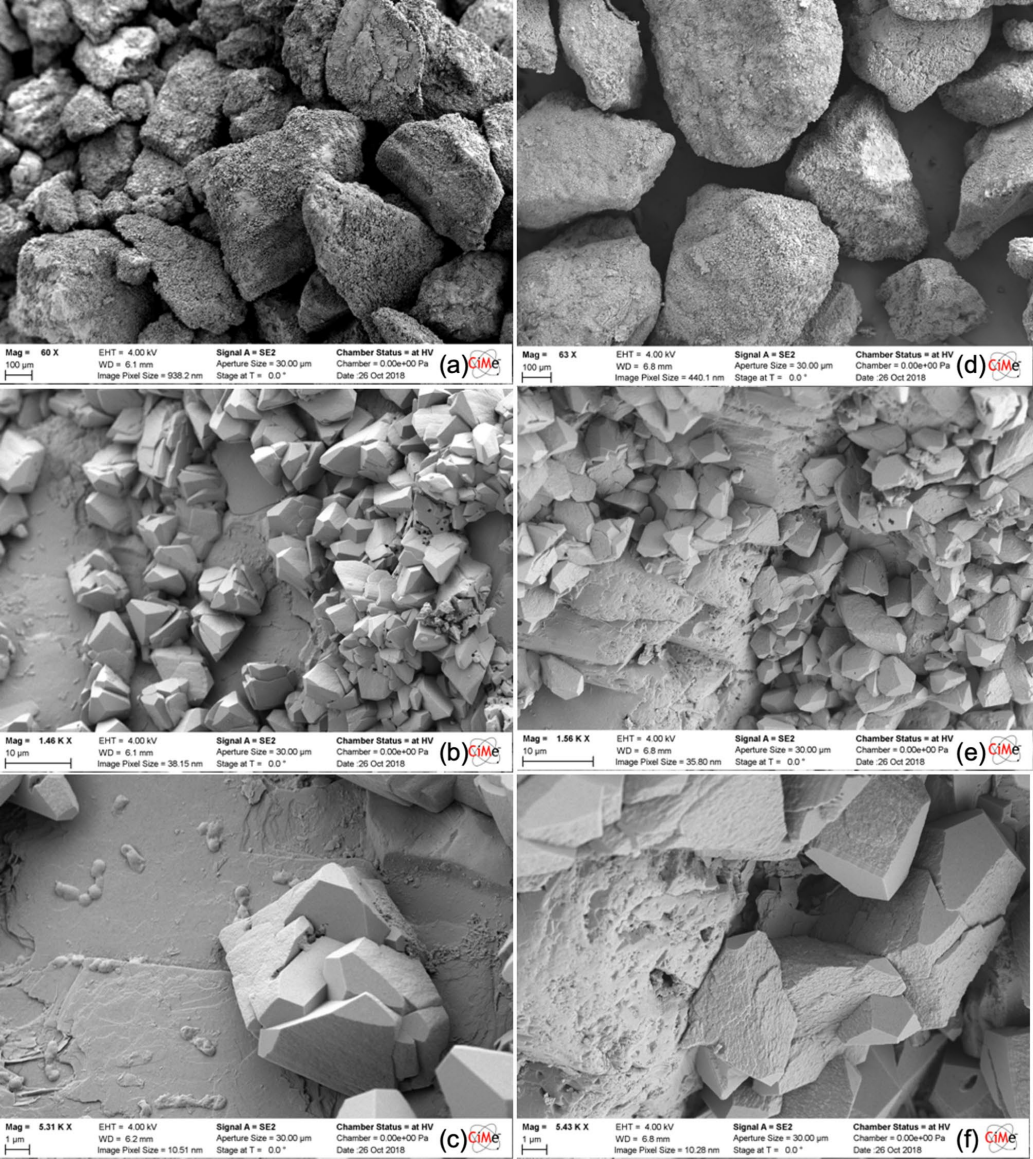
SEM observations on the surface of bio-cemented sand samples which were obtained after MICP (a to c) and EA-MICP (d to f) using CaL_2_ as reactive species.

CaL_2_-based MICP and EA-MICP treatment yield similar patterns in the textural characteristics of the precipitated CaCO_3_ crystals. Extensive sand grain coverage is captured in Fig. 10a,d. Observation of single crystals in Fig. 10b,c,e,f reveal crystals which exhibit well-faceted, rhombohedral morphologies with EA-MICP yielding slightly elongated particles with larger surfaces. This elongated form can be explained based on the hypothesis of the beneficial role of DC which can induce a more structured and elongated crystallization process for overgrown crystals.

**Figure 11:**
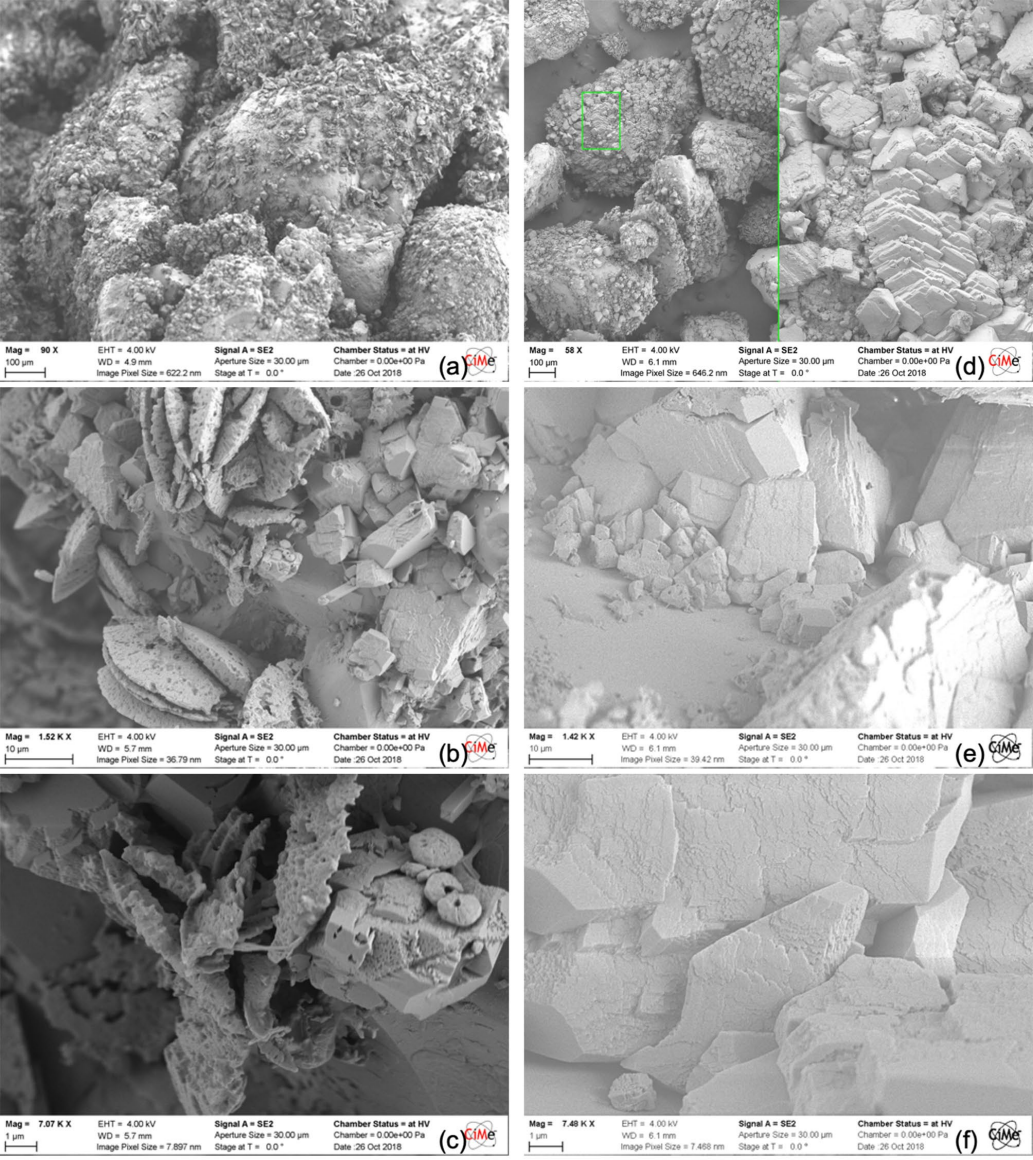
SEM observations on the surface of bio-cemented sand samples which were obtained after MICP (**a**–**c**) and EA-MICP (**d**–**f**) using CaAc_2_ as reactive species.

The more pronounced discrepancies between the MICP and EA-MICP treatments were captured for the case of CaAc_2_-based treatments in “Crystallography of MICP and EA-MICP” section with aragonite and vaterite peaks identified in Fig. 7c for the traditional MICP phase. This result is further validated through the SEM observations shown in Fig. 11. Here, the MICP treatment yields rather inhomogeneous and multi-phasic CaCO_3_ precipitation pattern with a mixture of vaterite, aragonite and calcite covering sand grains. Contrarily, EA-MICP yields predominantly calcite with compact, crystalline planes which resemble those observed in Figs. 10e,f. No plate-like or oyster-like vaterite or aragonite nuclei are seen, and crystalline formations induced by EA-MICP appear to have less impurities and to result in a better coverage of the sand grain surfaces (Fig. 11a,d).

**Figure 12:**
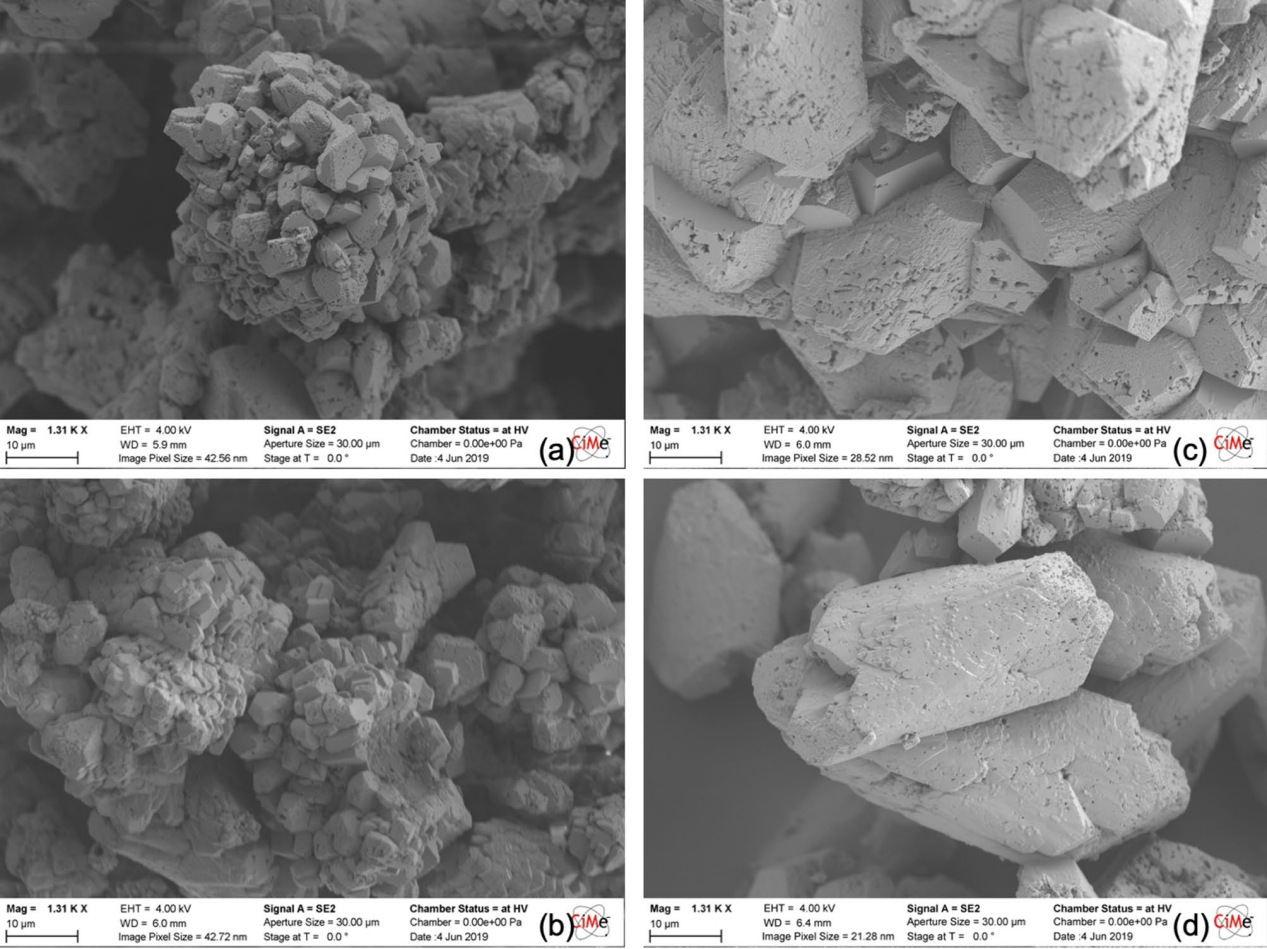
SEM observations revealing calcite crystals precipitated in solution using CaL_2_ under no-field (**a**) and applied field of 5 V (**c**) and CaAc_2_ under no-field (**b**) and applied field of 5 V (**d**) for the same magnifications.

Additional SEM observations on the texture and morphology of calcite crystals which precipitate under applied electric field are presented in Fig. 12 and compared with the characteristics of field-free samples. Crystals herein do not precipitate in sand columns but in intensively pre-electrolyzed solutions. Mineralized CaCO_3_ yielded using CaL_2_ and CaAc_2_ reveal morphologies which highlight the generally observed trend of crystals which grow larger planes, more stretched and elongated ones with less impurities or imperfections. We postulate that this “doped” morphology of crystals is due to the formation of a more hierarchical, or less random, distribution of ions under DC which modify drastically the chemical growth environment. Herein, it is thought that the intense electrolysis of the CaL_2_ and CaAc_2_ salt solutions, prior to the introduction of bacteria cells and mixing with urea, release in the chemical bath oxidative species of lactate and acetate ions. These act as chemical “guides” towards a directional growth of calcite crystals. The underlying mechanism which is the most intuitive is that the electrochemically generated species lower the specific planes of calcite crystals which then start growing faster than others and consequently induce a directed growth pattern. This finding implies that the effect of EA-MICP extends from influencing the calcite distribution and homogeneity across flow paths towards impacting the microstructural and mechanical properties of the precipitated nuclei according to the involved electrochemical reactions and generated sub-products.

## Conclusions

This work introduces the concept of EA-MICP and presents a series of benefits and limitations related to its application for soil bio-improvement. The complex reactive-transport phenomena involved in soil bio-cementation are herein understood as a bio-geo-electro-chemical system which can be influenced by the application of direct currents which induce electromigration of bacterial cells and dissolved solutes. Results present the effects of applied DCs of varying force and polarity on the distribution of CaCO_3_ crystals when precipitation occurs using CaCl_2_, CaAc_2_ and CaL_2_ as Ca^2+^ sources. EA-MICP has a detrimental effect when using CaCl_2_ for inducing bio-cementation due to the hypochlorination which destroys cells and creates acidic fronts that can dissolve carbonate species. However, when DCs apply in EA-MICP systems based on CaAc_2_ and CaL_2_, enhanced crystalline morphologies and CaCO_3_ distributions are obtained. Mineralized binders, grow and stretch their planes following more hierarchical patterns resulting in “doped” crystals. This result is validated using SEM observation of textural characteristics and XRD for determining crystallographic patterns or micro-CT for an in-depth description of the CaCO_3_ distribution across sand specimens. This improved structure and distribution is expected to influence the engineering properties of calcite bonds and the greater properties of the bio-cemented geo-material. The work overall introduces a new concept for MICP which sets new groundwork for developments and optimization in the field of soil bio-improvement in sands or finer materials with more active electro-chemical surfaces such as clays. We propose that the demonstrated, distinctive formation mechanisms of CaCO_3_ species under the presence of electric fields or varying chemical surface conditions, result of the various Ca^2+^ sources, are of key importance towards understanding and controlling the crystalline properties and thus the mechanics and deformation characteristics of the resulted, bio-cemented geo-materials.
